# Istradefylline to Treat Patients with Parkinson’s Disease Experiencing “Off” Episodes: A Comprehensive Review

**DOI:** 10.3390/neurolint12030017

**Published:** 2020-12-08

**Authors:** Amnon A. Berger, Ariel Winnick, Alexandra Welschmeyer, Alicia Kaneb, Kevin Berardino, Elyse M. Cornett, Alan D. Kaye, Omar Viswanath, Ivan Urits

**Affiliations:** 1 Department of Anesthesiology, Critical Care, and Pain Medicine, Beth Israel Deaconess Medical Center, Harvard Medical School, Boston, MA 02215, USA; amnonab@gmail.com; 2 Soroka University Medical Center and Faculty of Health Sciences, Ben Gurion University of the Negev, Beer-Sheva 8410501, Israel; ariel.winnick@gmail.com; 3 School of Optometry, University of California, Berkeley, CA 94704, USA; 4 Department of Anesthesiology, Georgetown University School of Medicine, Washington, DC 20007, USA; afw39@georgetown.edu (A.W.); ack75@georgetown.edu (A.K.); kmb364@georgetown.edu (K.B.); 5 Department of Anesthesiology, LSU Health Shreveport, Shreveport, LA 71103, USA; akaye@lsuhsc.edu (A.D.K.); viswanoy@gmail.com (O.V.); ivanurits@gmail.com (I.U.); 6 Department of Anesthesiology, University of Arizona College of Medicine—Phoenix, Phoenix, AZ 85004, USA; 7 Department of Anesthesiology, Creighton University School of Medicine, Omaha, NE 68124, USA; 8 Valley Anesthesiology and Pain Consultants—Envision Physician Services, Phoenix, AZ 85004, USA; 9 Southcoast Health, Southcoast Physicians Group Pain Medicine, Wareham, MA 02571, USA

**Keywords:** parkinsonism, levodopa, carbidopa, neurodegenerative, catechol-o-methyl transferase (COMT)

## Abstract

Parkinson’s disease (PD) is a common neurodegenerative disorder that leads to significant morbidity and disability. PD is caused by a loss of dopaminergic, cholinergic, serotonergic, and noradrenergic neurons in the central nervous system (CNS), and peripherally; the syndromic parkinsonism symptoms of movement disorder, gait disorder, rigidity and tremor are mostly driven by the loss of these neurons in the basal ganglia. Unfortunately, a significant proportion of patients taking levodopa, the standard of care treatment for PD, will begin to experience a decrease in effectiveness at varying times. These periods, referred to as “off episodes”, are characterized by increased symptoms and have a detrimental effect on quality of life and disability. Istradefylline, a novel adenosine A2A receptor antagonist, is indicated as a treatment addition to levodopa/carbidopa in patients experiencing “off episodes”. It promotes dopaminergic activity by antagonizing adenosine in the basal ganglia. This review will discuss istradefylline as a treatment for PD patients with off episodes.

## 1. Introduction

Parkinson’s disease (PD) is an increasingly prevalent neurodegenerative condition, second only to Alzheimer’s and steadily rising in prevalence, as many nations see their aging populations increase [1,2]. It may present with a variety of cognitive, motor, and behavioral symptoms. The primary pathology is the absence of dopaminergic neurons in the basal ganglion [3]. The estimated lifetime risk of Parkinson’s disease in the United States is 2% for men and 1.3% for women aged 40 years [4]. Several studies have indicated a small male predominance in the occurrence of PD, with ratios ranging from 1.3 to 2 [1,4], and the onset of disease can be sooner in men than in women [5]. In both men and women, incidence increases with age, beginning at age 50 and increasing to a peak at age 80 [4]. It is estimated that in 2020, there will be 930,000 individuals with a diagnosis of Parkinson’s Disease [6]. 

Many patients with PD experience cycles of deteriorating symptoms called “off-episodes.” These episodes may occur unexpectedly and unpredictably, affecting the quality of life of patients significantly [3]. These episodes are due in part to chronic levodopa therapy, which is one of the disease’s most common treatment methods. These episodes consist of both motor, non-motor, posture, and autonomic symptoms [3,7]. Patients with off-episodes also report anxiety about impending symptoms [3].

This paper provides a comprehensive review of the current literature involving istradefylline, an adenosine A2A-receptor antagonist, for the treatment of off-episodes in PD. It discusses the background, epidemiology, and prevalence of PD and describes the phenomenon of off-episodes. We will also examine the current therapeutic options that exist for PD and discuss more thoroughly the properties of istradefylline and its potential value and use in treating Parkinsonian off-episodes.

## 2. Experimental Section

### 2.1. Parkinson’s Disease

PD is the second most common neurodegenerative disorder [1]. It is a chronic disease that manifests with motor, cognitive, and behavioral symptoms that stem from loss of dopaminergic neurons in the basal ganglia [8]. There is currently no cure for PD, but there are a variety of therapeutic options that target both motor and non-motor features of the disease. 

#### 2.1.1. Epidemiology

PD is rising in global prevalence. In 2016, there were over 6 million cases of PD globally, which was more than two times higher than the number of cases reported worldwide in 1990 [2]. In the United States, the lifetime estimated risk of PD is 2% in men and 1.3% in women aged 40 [4]. A slight predominance in men has been reported by several studies, and this trend may become more pronounced with increasing age [9]. 

#### 2.1.2. Pathophysiology

PD is caused by a deficiency of dopamine in the brain driven by loss of dopaminergic neurons, particularly in the substantia nigra [6]. Dopamine is a neurotransmitter involved in important regulation and modulation of movement circuits. Through its actions on the dopamine receptors, D1 and D2, on striatal neurons, it is able to activate neurons in the direct pathway and inhibit neurons in the indirect basal ganglia pathways, respectively [8]. The direct pathway and indirect pathways influence the basal ganglia in opposite ways, with the direct pathway facilitating movement and the indirect pathway providing inhibitory modulation [8]. In PD, loss of dopaminergic neurons thus leads to increased inhibition of motor signaling systems, through inactivation of the globus pallidus pars interna, part of the direct pathway, and loss of inhibition of the subthalamic nucleus and globus pallidus pars externa, which make up the indirect pathway [8]. This theory is supported by findings that deep brain stimulation and lesions to these areas (substantia nigra (STN) and globus palladus (GPe)) produce some relief of Parkinsonian motor symptoms [8].

On autopsy, neurons in a Parkinsonian brain display the disease’s hallmark pathologic feature, which are cytoplasmic inclusions of alpha-synuclein protein, termed Lewy bodies [6]. PD is typically identified once the disease has begun to impact the substantia nigra pars compacta, which results in the motor symptoms (i.e., bradykinesia, rigidity) typically associated with clinical presentation of the disease [6]. Motor symptoms typically become present with loss of 50% of substantia nigra neurons [6]. However, the disease process begins earlier in the medulla and olfactory bulb, which causes symptoms such as sleep disturbances and reduced sense of smell, which may present before motor symptoms become recognizable [6]. The loss of dopaminergic neurons in PD is what contributes to its hallmark symptoms of bradykinesia and rigidity [10]. Autopsy reveals loss of melanin and dopamine-containing neurons in the substantia nigra. Lewy bodies, which are eosinophilic cytoplasmic inclusions, are also revealed in neurons [10].

Several subtypes of PD exist. These are often classified based upon clinical symptoms and presentation and include subtypes such as: Postural Instability and Gait Difficulty (PIGD); Tremor-Dominant; and Mixed Parkinson’s Disease. Additionally, PD may be specified as late-onset after 60 years of age (LOPD), which often has rapid onset of symptoms and disease progression, or young-onset from 20–40 years (YOPD), which follows a slower disease course. Age of onset is therefore recognized as an important factor in understanding disease progression, but clinical subtypes may also play a role, as tremor-dominant Parkinson’s often has a less-rapid onset than PIGD [10]. PIGD often presents with an increased rate of development of cognitive deficits, and patients with this subtype may be more likely to develop features of dementia [10]. In one study, it was observed that patients with LOPD were more likely to present with PIGD-type disease patterns [10].

The parkin gene mutation (*PARKN*) is associated with the subtype *PARK-2* type PD. This subset can include early-onset of symptoms such as dyskinesia, hallucinations, ataxia, and more. Thenganatt et al. identified the parkin gene mutation (*PARKN*) in one-third of patients with YOPD. Inherited forms of PD typically present at a younger age than idiopathic cases [9]. To date, 18 genetic loci related to Parkinson’s Disease have been identified [10]. Among autosomal dominant forms of Parkinson’s, the *LRRK2* mutation is most common [10].

#### 2.1.3. Risk Factors 

The vast majority of PD cases are not attributable to a specific genetic cause [4]. It is likely that the disease is due to contributions from both genetic and environmental factors [5]. However, it is apparent that age is the most important risk factor for the disease [2].

Exposure to environmental agents such as metals and pesticides has been linked to an increased risk of developing PD [6]. Conversely, smoking has been associated with a decreased risk of developing Parkinson’s Disease, and while some studies suggest that risk may decrease by 40%, true causality is still controversial, and most experts agree that the numerous detrimental health effects of smoking would outweigh a possible decreased risk of developing PD [2]. The risk reduction appears to be dependent on duration of smoking history; in one study, there was no additional benefit in patients who smoked more than 10 cigarettes per day [11].

Caffeine intake may also be inversely associated with risk of PD, possibly via protective mechanisms on dopaminergic neurons [12]. Finally, alcohol use has also been suggested to be inversely associated with risk of PD [11]. Similarly to smoking, duration of use was deemed to be more important, as the relationship did not increase in strength beyond moderate alcohol intake [11]. 

Presence of a first-degree relative with PD has been associated with nearly three times (2.8–2.9) greater risk of developing PD [11]. This risk does not appear to be mitigated by smoking, and the risk reduction association with tobacco is not as strong in these patients [11].

#### 2.1.4. Diagnosis and Clinical Presentation

Patients with PD can experience up to two decades of non-specific symptoms before motor symptoms set in [4]. These may include phenomena such as hyposmia (reduced sense of smell), constipation, and sleep disturbances [4].

In older patients, the incidence and diagnosis of PD may be underreported, due possibly to failure to seek medical treatment or to presence of other symptoms and diagnoses, such as dementia, which exclude a diagnosis of Parkinson’ s [4,13]. The diagnosis of PD is largely a clinical one, though imaging studies can be supportive [1]. Clinical diagnostic criteria commonly requires the presence of two of the following symptoms: presence of a resting tremor, bradykinesia, stiffness and rigidity, and postural instability [1,2].

In addition to motor symptoms, PD may include many components such as depression, urinary incontinence, orthostatic hypotension, sialorrhea, dementia, psychosis, erectile dysfunction, constipation, and sleep disturbances that may increase in prevalence with disease duration [14]. Thus, comprehensive care of patients with PD requires attending to motor and non-motor symptoms that can both significantly impact quality of life.

Imaging technologies that aid in the diagnosis of Parkinson’s include dopamine transporter single-photon emission computed tomography (DaT SPECT), which uses radioactive tracing substances to demonstrate reduced dopamine transporters and binding sites in Parkinsonian brains, specifically in the basal ganglia [6]. This imaging modality has high sensitivity and specificity (nearly 100%) for detecting neuronal loss. However, most often a diagnosis is able to be made clinically, and additional imaging is not needed [6]. 

### 2.2. Current Treatment Options

The motor symptoms of Parkinson’s disease are predominantly treated with dopamine-based medications including levodopa, dopamine agonists, and monoamine oxidase-B (MAO-B) inhibitors [6,15].

Levodopa is a precursor in dopamine synthesis. It has shown superiority over placebo in randomized controlled trials and has been considered the mainstay of Parkinson’s treatment for decades [16,17]. It is especially effective in the early stages of disease [17,18]. The PD MED trial found that patients initially treated with levodopa had benefits in mobility and activities of daily living compared to those treated with dopamine agonists or MAO-B inhibitors. Additionally, patients were more likely to discontinue the study medication or require add-on treatment with dopamine agonists or MAO-B inhibitors than with levodopa. While there was no difference among groups in motor fluctuations, patients on levodopa were more likely to develop dyskinesia [6,16,19]. Another drawback to levodopa is that patients often require higher and more frequent doses over time due to loss of efficacy [6]. Furthermore, levodopa has many associated side effects. After five years of treatment, approximately 40% of patients experience dyskinesia and motor fluctuations [16,20]. There is a higher risk for patients with early onset Parkinson’s, and a longer duration of treatment with higher doses [16,17]. Furthermore, impulse control disorders (e.g., compulsive spending or medication use, gambling, abnormal sexual behaviors) can occur, especially at high doses [16,21]. Punding, a subtype of impulse control where patients perform repeated purposeless actions (e.g., sorting and disassembling objects), occurs in 1.4%–14% of patients [16,22].

Dopamine agonists bind directly to the dopamine receptors in the striatum [16,23]. Their effectiveness for motor symptoms has been shown in randomized controlled trials and in a systematic review [16,24,25,26,27]. They are also useful in reducing “off” periods, where symptoms are not adequately controlled [28]. They are divided into ergot dopamine agonists (bromocriptine, cabergoline, and pergolide) and non-ergot dopamine agonists (ropinirole, pramipexole, and rotigotine) [16]. The National Institute for Health and Care Excellence guidelines recommend non-ergot dopamine agonists, as ergot dopamine agonists, which can cause retroperitoneal fibrosis and heart valve complications, require frequent monitoring [16,29]. A major side effect of all dopamine agonists is impulse control. More than 40% of patients treated with dopamine agonists experience an impulse control disorder [6,30]. Dopamine agonists are also more likely than levodopa to cause freezing, edema, somnolence, and hallucinations [28]. Other common adverse events include nausea, vomiting, constipation, edema, dizziness, and hypotension [16,27]. As a result of these adverse effects, patients are likely to stop taking the medication; yet 15%–20% of patients who discontinue therapy experience symptoms of withdrawal (e.g., irritability, anxiety, pain, diaphoresis). Thus, discontinuation of dopamine therapy poses its own challenges [6,31,32].

Since the MAO-B enzyme metabolizes dopamine, MAO-B inhibitors (selegiline and rasagiline) offer therapeutic benefit by increasing dopamine availability [16,23]. A systematic review found a small but significant motor improvement in early Parkinson’s disease for patients taking MAO-B inhibitors compared with placebo [16,33]. However, another systematic review found that more patients taking MAO-B inhibitors required add-on therapy compared with patients taking levodopa preparations or dopamine agonists [16,34]. There is conflicting evidence regarding the use of MAO-B inhibitors for “off” periods [28]. While dopaminergic side effects (e.g., nausea and vomiting) can occur, they occur less commonly with MAO-B inhibitors than with dopamine agonists [16,33]. 

Anticholinergic agents, such as trihexyphenidyl, are used occasionally but cautiously due to their adverse effects on cognition, especially in the elderly population [6,15,29]. Amantadine and beta-blockers have also been used historically, however, there are more efficacious options available and therefore they are not recommended as first line treatments [29]. Advanced Parkinson’s disease can be treated with catechol-o-methyl transferase (COMT) inhibitors, apomorphine, deep brain stimulation, MRI-guided focused ultrasound and intrajejunal levodopa gel; however, these are also not used as first line treatments [6,16]. Rather than as initial treatment, COMT inhibitors (e.g., entacapone, opicapone, tolcapone) are clinically useful for “off” periods by extending the half-life of levodopa [6,16,35]. Side effects of COMT inhibitors include dark-colored urine, exacerbation of levodopa side effects, and hepatotoxicity specifically with tolcapone [28].

Non-motor symptoms of Parkinson’s Disease are primarily treated with medications that address the specific symptom via neurotransmitters other than dopamine [6]. For example, rivastigmine (a cholinesterase inhibitor) is used for dementia and selective serotonin reuptake inhibitors are used for depression [36].

Overall, there is a deficit of sufficient research comparing different therapies for Parkinson’s disease [18]. None of the available treatment options are completely effective and they each pose side effects. An effective treatment strategy requires shared decision making that considers the patient’s desires alongside the risks and benefits of the various options [6]. The challenge for future therapies is to maximize clinical effectiveness while minimizing adverse events, a common hurdle for therapeutic interventions in medicine.

## 3. Results

### 3.1. Istradefylline

Istradefylline (trade name Nourianz^TM^) is an adenosine A2A receptor antagonist approved by the U.S. Food and Drug Administration (FDA) as an add-on treatment to levodopa/carbidopa for adult Parkinson’s disease patients experiencing “off” episodes [37]. It has shown efficacy in experimental primate and rodent models of Parkinson’s disease. Although there was only a modest effect of the drug alone, there was a robust improvement in motor function when combined with levodopa or dopamine agonists [38,39,40].

The FDA recommends an oral dose of 20 mg once daily with a maximum dose of 40 mg once daily for istradefylline. It can be taken with or without food. While patients with mild hepatic impairment (Child-Pugh Class A) do not need dosage adjustment, in patients with moderate hepatic impairment (Child-Pugh Class B) the recommended maximum dose is 20 mg daily and in patients with severe hepatic impairment (Child-Pugh Class C) the medication should be avoided. However, it has not been specifically studied in subjects with severe hepatic impairment [37]. No dosage adjustment is needed in patients with mild, moderate, or severe renal impairment (CrCL 15 mL/min or greater), but it has not been studied in patients with end-stage renal disease (CrCL < 15 mL/min). In patients who smoke 20 or more cigarettes per day, the recommended dosage is 40 mg once daily due to a decreased steady-state exposure of 38%–54% in this population. Based on animal data, it may cause fetal harm in pregnant patients; however, this has not been explored in humans. Currently, there are no absolute contraindications for use [37].

#### 3.1.1. Pharmacology

Istradefylline works as a selective antagonist of the adenosine A_2A_ receptors [41,42]. The adenosine A_2A_ receptors are primarily localized to the basal ganglia on the external surfaces of neurons in the indirect output pathway between the striatum, external globus pallidus, and substantia nigra [38,43,44]. The adenosine A_2A_ receptors are co-localized with the dopaminergic D2 receptors in the indirect pathway, which allows for antagonistic interactions between adenosine and dopamine [44]. Adenosine A_2A_ receptor activation decreases the affinity of dopaminergic D2 receptors for dopamine agonists and subsequently decreases mobility [44,45]. Therefore, antagonism of the adenosine A_2A_ receptor has potential to improve mobility for Parkinson’s patients through enhancement of dopaminergic D2 receptor activation [44]. Furthermore, adenosine antagonists have a neuroprotective effect on the dopaminergic neurons that are affected in Parkinson’s [44]. Adenosine A_2A_ receptor activation also inhibits gamma-aminobutyric acid (GABA) release and GABA-ergic transmission in the striatum while enhancing GABA-ergic transmission in the globus pallidus, thus disturbing a delicate balance of neurotransmission in these areas [44]. This disturbance can be corrected by receptor inhibition with adenosine A_2A_ receptors such as istradefylline [44].

The pharmacokinetics of oral istradefylline are described as a two-compartment model with first-order absorption [42,46]. The maximum plasma concentration (C_max_) and the area under the plasma concentration-time curve (AUC) increase dose-proportionately in Parkinson’s disease patients and healthy subjects [42,47,48]. In healthy subjects, the half-life, apparent volume of distribution, and clearance are also dose-independent [42,48]. Median time to reach C_max_ is about 4 h under fasting conditions [37]. Steady-state is reached within two weeks of once daily dosing [37]. Total clearance is approximately 4.6 L/h with a mean terminal half-life (t_1/2_) of 83 h [37]. Istradefylline is primarily metabolized by cytochrome P450 (CYP) 1A1 and 3A4 [37]. With a 40 mg oral dose, approximately 48% is eliminated in feces and 39% is eliminated in the urine [37]. Istradefylline exposure is affected by smoking and the use of CYP3A4 inhibitors. The AUC during a dosage interval is reduced by 38% in smokers while increased by 35% when there is a CYP3A4 inhibitor present [42,46]. Istradefylline itself is both a weak inducer and inhibitor of CYP3A4, but does not induce nor inhibit CYP1A2 [37]. Importantly, coadministration of istradefylline with levodopa/carbidopa does not alter the pharmacokinetics of levodopa/carbidopa [42,49].

#### 3.1.2. Side Effects and Adverse Events

In the four randomized, double-blind, multicenter, placebo-controlled clinical trials, the rate of patients discontinuing the study drug for adverse reactions was 5% for istradefylline 20 mg, 6% for istradefylline 40 mg, and 5% for placebo [37]. The most common adverse events (frequency at least 5% and greater incidence than placebo) were dyskinesia, constipation, dizziness, nausea, hallucination, and insomnia [37].

### 3.2. Discussion: Istradefylline in Parkinson’s Treatment—Review of Clinical Trials

Overactivation of adenosine A2 receptors is important in the pathogenesis and potentiation of “off” episodes in patients with Parkinson’s Disease (PD). Recently, istradefylline, an adenosine A2 antagonist, has shown promise in preventing and minimizing these breakthrough episodes of dyskinesia. Through targeted modification of adenosine A2A receptors expressed in the basal ganglia, istradefylline counteracts the common side effects of levodopa treatment such as wearing off, dyskinesias, and on-off fluctuations with potential for additional neuroprotective effects [50,51]. Istradefylline is a safe and effective treatment which helps reduce “off” time and reverse motor disability during “on” time without provoking dyskinesia secondary to previous exposure to levodopa or dopamine agents [41,52,53].

A preliminary double-blind, placebo-controlled, proof-of-principle study evaluated the mechanism and function of KW-6002 (istradefylline) in the treatment of PD for patients on concurrent levodopa. This trial of 15 patients found that istradefylline prolonged the effective half-time of levodopa and improved cardinal parkinsonian signs, especially resting tremor [54]. Results of this study helped support the hypothesis that adenosine A2 receptors contribute to the motor symptoms of PD and identified istradefylline as a promising potential treatment target.

A 2003 double-blinded, randomized, placebo-controlled exploratory study evaluated the safety and efficacy of KW-6002 (istradefylline) in patients with levodopa-treated PD with both motor fluctuations and peak-dose dyskinesias. Participants were randomly assigned to treatment with placebo (*n* = 29), istradefylline 20 mg/day (*n* = 26), or istradefylline 40 mg/day (*n* = 28) for a duration of 12 weeks. Though no primary outcome measurement was prespecified, the authors evaluated daily change in “off” time from baseline as indicated by participants’ daily diary entries. Additionally, the Unified Parkinson’s Disease Rating Scale (UPDRS), Clinical Global Impression (CGI) were measured and were found to be unchanged across all three treatment groups. However, the authors found that participants assigned to istradefylline treatment experienced a significant reduction in the proportion of awake time spent in the “off” state compared to controls. Additional significant increases in “on” time with dyskinesia were noted in the istradefylline groups versus placebo. While istradefylline was generally well-tolerated, the most common adverse event was nausea [55]. The preliminary results of this study demonstrated the safety and efficacy of istradefylline for reducing “off” time and increasing “on” time with dyskinesia.

Similarly, Mizuno et al. (2010) performed a phase III randomized controlled trial (RCT) of patients with PD to evaluate the clinical efficacy and safety of KW-6002 (istradefylline) 20 mg and 40 mg relative to placebo in controlling “off” episodes amongst patients taking levodopa over a 12-week period. Participants were required to experience at least two hours of “off” time daily, classified as PD Stages 2 to 4, despite receiving at least three doses of L-dopa/decarboxylase inhibitor (DCI) per day for a daily total of at least 300 mg and while on anti-parkinson treatment for at least four weeks prior to randomization. Of the 363 participants randomized to treatment groups, 357 (98.3%) were included in the full analysis: 20 mg/day istradefylline (*n* = 115), 40 mg/day istradefylline (*n* = 124), placebo (*n* = 118). The primary outcome of interest was daily change in “off” time from baseline based on daily participant diary entries documenting time spent in the following states: Asleep, OFF state, ON state without dyskinesia, ON state with non-troublesome dyskinesia, and ON state with troublesome dyskinesia. Additionally, the UPDRS Part III subscale score and CGI-Improvement (CGI-I) were evaluated at the study endpoint. It was found that patients who received both 20 mg/day and 40 mg/day of istradefylline had significantly less OFF time compared to those who received placebo. Additionally, those who received 40 mg/day istradefylline had significantly increased ON time with troubling dyskinesia compared with placebo. The UPDRS Part III scores improved significantly for participants receiving both doses of istradefylline relative to placebo; the improved UPDRS Part III scores reflect improvements in motor function during ON states. The percentage of participants with improvements in CGI were higher in the 20 mg/day and 40 mg/day istradefylline groups compared with placebo, but not by a significant degree. Regarding safety, during this trial no deaths were reported. Rates of treatment emergent adverse events (TEAEs) were comparable across all three treatment groups, the most common of which was dyskinesia. The results of this trial were promising and reproducible; a subsequent 12-week RCT by Mizuno and Kondo (2013) of 373 subjects similarly found significantly reduced OFF time with both istradefylline 20 mg/day and 40 mg/day compared with placebo [56]. Overall, the results of these trials demonstrate the clinical efficacy istradefylline at both 20 mg/day and 40 mg/day doses in improving OFF time in patients with PD on levodopa while maintaining a good safety profile [57]. These dose-related results are further supported by a 2018 study in Japan which assessed occupancy of adenosine A2A receptors by istradefylline at differing doses. The authors found that sufficient adenosine A2A receptor occupancy was obtained with both 20 mg or 40 mg istradefylline [58]. The receptor occupancy by drug binding acts as a surrogate metric to assess adequacy of dosing regimens.

The 6002-US-005 double-blind, randomized, multicenter clinical trial investigated the efficacy of KW-6002 (istradefylline) in patients with PD treated with levodopa experiencing prominent wearing-off motor fluctuations. The primary measure of efficacy was percentage of daily awake “off” time measured by daily home diary entries; secondary outcomes of interest included “on” time, UPDRS, CGI improvement of illness score, and adverse events. “On” time was further classified as with or without dyskinesia and troublesome versus non-troublesome dyskinesia. 172 subjects met eligibility criteria and completed the trial which included the following: diagnosis of idiopathic PD, Hoehn and Yahr scale severity of 2 to 4, levodopa responsiveness for at least one year, and wearing off of antiparkinsonian benefit for at least 2 h daily. Participants were randomly assigned to receive istradefylline 40 mg/day (*n* = 114) or placebo (*n* = 58) for a duration of 12 weeks, while simultaneously taking routine levodopa. Those who received istradefylline 40 mg/day had significantly decreased daily awake “off” time compared with those who received placebo. The istradefylline treatment group also had significantly increased “on time with dyskinesia” than controls; this increase in “on” time correlated with the decrease in “off” time. Low and comparable rates of adverse events were observed in both groups; however, the most commonly reported events were dyskinesia, dizziness, insomnia, nausea, and accidents involving falls [59]. This trial demonstrated the favorable safety profile and promising efficacy of istradefylline in reducing “off” time and increasing “on” time in levodopa-treated patients with PD.

The 6002-US-006 double-blind RCT sought to further evaluate the safety and efficacy of istradefylline in the treatment of levodopa-treated PD for patients with motor complications. A total of 347 participants taking levodopa were randomized to the following treatment groups and included in the intention-to-treat analysis: istradefylline 20 mg/day (*n* = 152), istradefylline 60 mg/day (*n* = 126), or placebo (*n* = 69) over a 12-week period. Participants included in the trial had PD and were all levodopa-responsive for at least one year with an average of at least 2 h/day of OFF time. The primary outcome of interest was change in the percentage of daily time spent in the OFF state. Additional outcomes measured included ON time, UPDRS, and CGI. Drug safety was monitored through clinical labs, electrocardiograms, vital signs, physical/neurological examinations, and adverse events. Using an intention to treat analysis, the authors found that both istradefylline 20 mg/day and 60 mg/day resulted in a significant decrease in OFF time compared with placebo. Interestingly, there were no significant differences in UPDRS and CGI between groups after the 12-week treatment period. Though istradefylline was generally well tolerated, the most common adverse events were dyskinesia, nausea, dizziness, and hallucinations. These adverse events occurred at comparable rates across all groups [60]. Evidence from this RCT supports the use of istradefylline to improve OFF rates amongst patients with PD treated with levodopa.

The KW-6002-US-018 phase III randomized double-blind, placebo-controlled parallel-group study also investigated the efficacy of istradefylline in reducing levodopa-related motor complications. A total of 610 participants with PD were randomized to receive 12-weeks of either istradefylline 10 mg/day (*n* = 149), 20 mg/day (*n* = 144), 40 mg/day (*n* = 145), or placebo (*n* = 146). The authors measured change from baseline in percentage of awake time spent in the OFF state as measured by daily patient diary logs and change in UPDRS Part III score, which were evaluated using an intention-to-treat analysis. Surprisingly, the results of this study differed from prior RCTs. The amount and percentage of OFF time was comparable across all treatment groups; however, there was a modest but significant improvement in UPDRS score in the istradefylline 40 mg/day group compared with placebo. The most common reported adverse events were dyskinesia and insomnia [61,62]. In contrast to prior studies, adjunctive istradefylline in this study did not meet the primary goal of reducing OFF time in patients with motor fluctuation, thus warranting future studies to better classify its use.

A meta-analysis was performed to further assess efficacy and safety of adjunctive istradefylline in patients with levodopa-treated PD. Pooled data from five RCTs found a significant reduction in daily awake time spent in the OFF state and improvements in UPDRS Part III score in the ON state when taking istradefylline compared to placebo. No significant difference in UPDRS part III between istradefylline 20 mg/day and 40 mg/day were observed; however, only participants who received istradefylline 40 mg/day showed significant improvement in dyskinesia compared with controls. Comparable rates of adverse events were found across all groups, thereby illustrating the relative safety of istradefylline compared to placebo [63]. The results of the aforementioned meta-analysis highlight the efficacy and safety of istradefylline in improving motor fluctuations in patients with PD.

A subsequent meta-analysis compiled and analyzed data from seven RCTs to investigate effects of istradefylline on daily OFF time and UPDRS Part III score in patients with PD with motor fluctuations. A total of 2205 patients were included in the analysis. Istradefylline doses of 20 mg/day, 40 mg/day, and 60 mg/day were found to improve daily OFF time and UPDRS Part III scores significantly compared with placebo. Furthermore, there was no observed difference in rates of treatment-emergent adverse events between treatment and placebo groups. The most common adverse events were dyskinesia (istradefylline 18.9%, placebo 11.1%) and nausea (istradefylline 8.3%, placebo 5.3%). Though the evidence presented is promising and suggests that istradefylline can serve as a beneficial augmentation drug to patients on levodopa or other anti-PD therapies, the number of existing RCTs is limited. Additionally, many of the RCTs included in this study only assess short-term outcome (e.g., 12 week treatment period), do not assess efficacy of different doses, and fail to investigate istradefylline as a monotherapy [64]. Future RCTs should aim to address these limitations and expand the study period to include long-term effects.

Similarly, results from a different meta-analysis of six RCTs found that istradefylline 40 mg/day decreased OFF time and improved motor symptoms in homogeneous studies while istradefylline 20 mg/day decreased OFF time and improved motor symptoms with heterogeneity in the analysis. Interestingly, it was found that dyskinesia was worsened by istradefylline in homogeneous studies. Overall, these results are consistent with prior meta-analysis findings; however future studies are warranted due to variability in the findings of dyskinesia with istradefylline treatment.

A 2010 open-label, multicenter, safety and tolerability trial was performed in the United Kingdom and revealed promising results. A total of 496 subjects with PD on levodopa for at least one year were recruited and assigned to receive open-label istradefylline 40 mg/day for two weeks followed by a dose ranging from 20–60 mg/day thereafter with a mean exposure of 25 weeks. Participants assigned to Group I (*n* = 315) were those who had completed double-blind treatment with istradefylline within 15 days before entering this study. Those in Group II (*n* = 181) were considered either washed-out from istrafedylline therapy (greater than 15 days since last dose) or naïve to istradefylline (previously in placebo group). Though no significant reduction in OFF time was noted in Group I, the mean decrease in OFF time for participants in Group II was significant and showed evidence of consistent improvement over a 52 week duration. However, this data should be interpreted with caution due to poor participant retention (23%) [65]. The promising yet poorly quantified outcomes of this study have paved the way for additional open-label studies to come.

A subsequent single-arm, open-label, prospective, multicenter study investigated the use of istradefylline for gait disorders with freezing of gait in PD. A total of 31 patients with levodopa-treated PD with a history of gait freezing were recruited. Participants received oral istradefylline 20 mg/day for the first four weeks followed by 20 mg/day or an increased dose of 40 mg/day for eight weeks if no tolerability issues occurred for a total treatment period of 12 weeks. Outcomes of interest included changes in total gait-related scores of the Part II/III UPDRS and Freezing of Gait Questionnaire (FOG-Q). Additionally, gait was analyzed by portable gait rhythmogram. At week 4, participants had significantly improved UPDRS Part II gate-related items total score which was sustained until week 12. FOG-Q total scores remained similar at week 4 as compared to their baseline score; however, this score was significantly decreased at week 12. Participants showed significantly increased overall movement per 48 h as measured by portable gait rhythmogram. Adverse events were reported in 7 of 31 participants and included, but was not limited to, the following: dyskinesia, cellulitis, pneumonia, insomnia, diarrhea, and headache [66]. All participants continued the study drug due to mild severity of adverse events. Though larger RCTs are warranted, this study demonstrated the relative efficacy and safety of istradefylline in improving gait disorders in PD patients complicated with freezing of gait.

Another study found that istradefylline enhances the treatment efficacy of suboptimal doses of levodopa (L-DOPA) and threshold doses of dopamine agonists amongst 1-methyl-4-phenyl-1,2,3,6-tetahydropyridine (MPTP)-treated marmosets. Administration of istradefylline significantly increased locomotor activity and increased ON time when co-administered with suboptimal L-DOPA and threshold ropinirole compared with ropinirole or control groups alone. Interestingly, istradefylline co-administration further improved motor disability and reduced OFF time more so than the improvement achieved with optimized dopaminergic therapy. The findings of this study demonstrate that supplementation of suboptimal L-DOPA and dopamine agonist anti-parkinson treatments with istradefylline can avoid dose escalation and help avoid the subsequent instances of adverse events seen with higher dopaminergic therapy doses [67]. Furthermore, evidence from a retrospective study of fifteen patients found that istradefylline 20 mg/day for 12 weeks resulted in shorter daily OFF times, lower required doses of L-DOPA, and higher ON-UPDRS Part III scores than was shown in previous RCTs [68]. These findings are limited by small sample size and lack of randomization or controls; however, it suggests that istradefylline 20 mg/day can improve motor fluctuations in patients with PD who experience mild-wearing off and potentially allow for reductions in necessary L-DOPA dosage.

Given the increasing evidence pointing to the success of istradefylline in treatment of OFF episodes in patients with PD, a study evaluating population pharmacodynamics of istradefylline was conducted. This study compiled data from six phase II/III clinical trials to characterize the pharmacokinetic-pharmacodynamic relationships for the following: percentage of OFF time, dyskinesia and dizziness, and dosages of istradefylline. It was found that the typical maximum decrease in OFF time percentage was 5.79% and that the probability of dyskinesia and dizziness most often plateaus at the 40 mg/day istradefylline dosage while the probability of nausea is expected to rise with increased doses [69]. Results of this analysis indicate that a starting dose of istradefylline is optimized between 20 mg/day to 40 mg/day in order to maximize OFF time while minimizing unwanted events such as dyskinesia, dizziness, and nausea.

The utility of istradefylline is not limited to treatment of “OFF” episodes for patients with PD. Recent literature has explored the application of istradefylline in treatment of postural deformities seen in PD. One of the most common causes of postural deformities in patients with PD is medications (e.g., dopamine agonists). A recent three-month open-label study investigated the potential use of istradefylline as treatment for postural abnormalities in 21 levodopa-treated patients with PD. The authors found that the subitem score of posture on the Movement Disorder Society revision of UPDRS part III significantly improved with istradefylline treatment; however, the changes in score of posture did not correlate with changes in other items on the MDS-UPDRS part III measures, qualitative questionnaires, or sleep scale changes [70]. An additional study of four patients with PD-associated postural deformities on dopamine agonists were treated with istradefylline. Three of the four patients were found to have clinical improvements in posture upon discontinuing the dopamine agonist while continuing istradefylline treatment [71]. In contrast to the above findings, a case report of a 68-year-old male with PD observed that istradefylline incidentally induced reversible plethorothotonus, a postural phenomenon characterized by abnormal lateral trunk flexion. The plethorothotonus was first observed four months after initiating istradefylline to treat wearing-off episodes. Subsequent discontinuation of istradefylline resulted in gradual improvement of plethorothotonus symptoms over the following four months [72]. Though this case documents only one patient with this adverse effect, posture-related adverse events related to istradefylline must certainly be further evaluated.

Another study illustrated the use of istradefylline in improving depression-like symptoms in patients with PD in an open-label trial. Thirty patients with PD were enrolled and received 20 mg istradefylline for four weeks followed by 40 mg for eight weeks. After treatment with istradefylline, participants had significantly improved Snaith-Hamilton Pleasure Scale Japanese version (SHAPS-J), Apathy scale, and Beck Depression Inventory-2nd edition (BDI-2nd edition) [73]. Though the results of this study are limited by small sample size and lack of randomization, the preliminary findings are promising. Additional evidence supports the beneficial neuropsychiatric profile of istradefylline given its ability to reduce OFF time while retaining the positive effects on mood and cognition seen with dopamine agonists [74]. Positive effects of istradefylline on PD-related cognitive function have also been documented. Murine models have shown that overexpression of adenosine A2A receptors cause impairment in working memory and thus support the hypothesis that adenosine A2A antagonists such as istradefylline can have a role in improving short-term memory and may enhance long-term memory in patients with PD [75]. Future directions should include more robust RCTs to further evaluate istradefylline’s additional utility in treating PD-related mood and cognitive symptoms. Additional preliminary evidence suggests that istradefylline may favorably alter the course and symptoms of PD [76]. Through prevention of underlying dopaminergic neuron degeneration, istradefylline confers beneficial neuroprotection.

In addition to its efficacy in treating motor symptoms of PD, istradefylline has also demonstrated promise in the treatment of antipsychotic-induced oral tremor. Antipsychotics, such as pimozidine, are known to cause parkinsonian-like symptoms such as akinesia and tremor. A controlled study using rat models compared istradefylline and tropicamide, a muscarinic antagonist, in the treatment of pimozidine-induced oral tremor. Results showed that istradefylline significantly reduced ventrolateral neostriatum overactivation and improved symptoms of pimozidine-induced oral tremor in subjects on pimozidine. Interestingly, tropicamide increased ventrolateral neostriatum activation yet achieved comparable improvement in symptoms. Thus, though it has a different mechanism of action, istradefylline proved to be as effective a treatment as tropicamide for pimozidine-induced oral tremor [77]. Another study in rats found similar results in the treatment of antipsychotic-induced tremulous jaw movements [78]; the relative success of istradefylline in the treatment of antipsychotic-induced oral/jaw tremor acts as a surrogate for its utility in producing antiparkinsonian effects. Studies as such this also serve to expand the utilization of istradefylline to treatment of both idiopathic and antipsychotic-induced parkinsonian symptoms.

With known evidence of the efficacy of antiparkinsonian drugs against restless leg syndrome (RLS), a 2007 study investigated the effectiveness and tolerability of istradefylline for RLS amongst five female patients. This prospective trial was designed such that participants with known moderate to severe idiopathic RLS were given a daily oral 80 mg dose of istradefylline for six weeks. Of the five subjects, three (60%) noted improvement in periodic limb movement and international RLS rating scale compared with baseline when treated with istradefylline [79]. Though the sample size was limited and definitive benefits could not be identified, the result of this trial were encouraging, and certainly warrant the need for future studies. An alternate study of four patients investigated daytime sleepiness after treatment with istradefylline in the evening. While the sample size was small and larger RCTs are needed, this preliminary case report found that patients experienced daytime sleepiness between two weeks to three months after initiating evening istradefylline treatment due to a presumed caffeine-like arousal effect which altered patients’ sleep quality. The sleepiness resolved when istradefylline dosing was changed from evening to morning [80]. Other evidence points to the clinical utility of istradefylline in treating lower urinary tract symptoms seen in PD. Thirteen male patients with PD were enrolled to receive istradefylline 20 mg/day and were evaluated for changes in lower urinary tract symptoms after 4, 8, and 12 weeks of therapy. Results showed that patients participants had significant decreases in International Prostate Symptom Score (IPSS) at 8 and 12 weeks, incomplete emptying at 4, 8, and 12 weeks, urgency at 4 and 12 weeks, and improved quality of life at 12 weeks. Overactive Bladder Symptom Score (OABSS) at 4 and 12 weeks, nocturia at 8 and 12 weeks, and urgency at 4 and 12 weeks significantly improved after istradefylline treatment. No adverse urological effects were observed [81]. This preliminary evidence holds promise for other future uses of istradefylline in improving motor dysfunction for patients with PD, such as reducing urinary tract symptoms.

Recent research directions have included investigation of purinergic signaling in modulation of dopaminergic neurotransmission. In addition to the key role of adenosine A1 and A2A receptors, purine nucleotides have been implicated in the regulation of dopamine neurotransmission, thus contributing to the motor dysregulation seen in in PD. A 2015 study found that purinergic P2X1 receptors mediate accumulation of alpha-synuclein, a Lewy-body enriched protein, involved in PD pathophysiology [82]. These purinergic receptors are potential future treatment targets and should be further investigated.

Due to the persistent nature of motor fluctuations and OFF states in patients treated for PD, many studies are under way to develop novel treatment strategies to address these negative symptoms. In addition to istradefylline, repetitive transcranial magnetic stimulation (rTMS) has recently been under investigation as a potential adjunctive treatment for patients with PD. rTMS has been shown to have a positive effect on the motor function of patients with PD. A 2015 comparative randomized clinical trial randomly assigned 132 patients with PD in China to receive 20 mg/day istradefylline plus sham-rTMS (Group I), 40 mg/day istradefylline plus sham-rTMS (Group II), placebo pluz 1Hz rTMS (Group III), or placebo plus 10 Hz rTMS (Group IV) for 12 weeks. Outcomes of interest included UPDRS Part III score and CGI-I. The authors found that there was no significant difference in UPDRS part III scores or CGI-I before and after treatment across all groups. No severe adverse events were reported. Thus, the results indicate that istradefylline and rTMS had comparable efficacy and tolerability. Interestingly, this study found that istradefylline 40 mg/day resulted in a non-statistically significant improvement in OFF time compared with istradefylline 20 mg/day [83]. These dose-related findings should be further evaluated in future studies.

Based on the existing evidence, istradefylline appears to be an effective and safe adjunctive treatment for patients with PD experiencing motor fluctuation. It most commonly helps patients achieve decreased awake time spent in the OFF state, by up to 25% in some studies, and improves UPDRS Part III scores when used in combination with levodopa or dopamine agonists [61]. Importantly, dosing ranges for the aforementioned clinical trials range from 20 mg/day to 80 mg/day, thus posing as a potential confounding factor when interpreting the data [84]. Future clinical trials should be aimed at expanding upon the current data to better characterize treatment dosing, efficacy, and adverse events. See Table 1 and Table 2.

## 4. Conclusions

PD is a common source of morbidity and disability. It is the second most common neurodegenerative disorder and has an increasing prevalence; in 2020, it is estimated that 930,000 individuals will live with PD in the USA alone. The pathophysiology is most likely through the loss of dopaminergic, serotonergic, noradrenergic and cholinergic neurons and decreased signaling, mostly in the basal ganglia but also in the periphery. Clinical presentation includes the classic parkinsonism signs including movement disorders, rigidity, postural instability, gait disorders and tremor, but also include urinary and gastrointestinal symptoms, sleep disorders, and cognitive and psychiatric perturbances. 

Traditional treatment is aimed at replacing these missing neurotransmitters and compensating for the neuronal death. Levodopa, aimed at increasing dopaminergic signaling in the basal ganglia, is a cornerstone of treatment and is usually included in the treatment regimen. Unfortunately, as the course of illness develops, patients begin to experience “off episodes” as the effects of levodopa wane between doses. There is great variation in the appearance of these episodes, and up to 30% of patients will start experiencing them as early as several weeks into treatment; almost every patient will report “off episodes” by 10 years of treatment. These “off” periods are a source of disability and greatly reduce patient quality of life.

Istradefylline is an adenosine A2A receptor antagonist that is FDA approved as a combination therapy with carbidopa/levodopa in patients experiencing “off episodes”. The recommended dose is 20 mg–40 mg once daily, and should not exceed 20 mg in patients with moderate hepatic insufficiency (Child-Pugh B); it should be avoided in patients classified as Child-Pugh C altogether, mostly due to lack of evidence of its safety. It was not studied in patients with end-stage renal disease, though it is not contra-indicated in that population. By antagonizing adenosine in the indirect pathway, Istradefylline tips the balance towards increased dopaminergic activity in the basal ganglia and thus synergizes with levodopa, decreasing motor symptoms and targets “off episodes”.

Several clinical trials evaluated the safety and efficacy of istradefylline in combination with levodopa/carbidopa and demonstrated significantly increased efficacy of treatment. With both approved doses, daily 20 mg or 40 mg, patients experienced less side effects from levodopa treatment (namely, dyskinesia), increased efficacy and improved activity (“ON”) times from levodopa, and reduced length and incidence of “off episodes”. Importantly, istradefylline was also shown to improve gait, decrease psychiatric symptoms and improve overall quality of life.

The most common side effects in these trials were dyskinesia, nausea, dizziness, and hallucinations; however, these side effects were not more common than in the placebo group and are likely not directly caused by istradefylline. Post-marketing surveillance will be required to assess for rarely occurring side effects and the true magnitude of adverse events.

## Figures and Tables

**Table 1:** Clinical Efficacy and Safety.

Author (Year)	Groups Studied and Intervention	Results and Findings	Conclusions
Jimenez et al. (2003)	Fifteen patients with Parkinson’s Disease (PD = on concurrent levodopa were randomly assigned to receive KW-6002 (istradefylline) or placebo for a duration of 6 weeks. Participants assigned to treatment groups received an initial 2 weeks of placebo followed by 2 weeks of istradefylline 40 mg/day then 2 weeks of istradefylline 80 mg/day. Assessments of motor function were based on the Unified Parkinson’s Disease Rating Scale (UPDRS) ratings.	Istradefylline, when given alone or combined with optimal levodopa infusion, had no effect on severity of PD. Istradefylline 80 mg/day when given with sub-optimal levodopa improved the antiparkinsonian response by 36% (*p* < 0.02) and showed 45% less dyskinesia than levodopa alone (*p* < 0.05). Concomitant administration of istradefylline with levodopa prolonged the efficacy half-life of levodopa by a mean of 47 min (*p* < 0.05). No adverse drug effects were observed.	Istradefylline may help mitigate motor symptoms in patients with PD treated with levodopa.
Hauser et al. (2003)	A total of 63 participants with levodopa-treated PD with motor fluctuations and peak-dose dyskinesias were randomly assigned to treatment with placebo (*n* = 29), istradefylline 20 mg/day (*n* = 26), or istradefylline 40 mg/day (*n* = 28) for 12 weeks. Twenty-four percent of participants assigned to placebo and 20% of those assigned to istradefylline withdrew from the study. Changes from baseline in daily “off” time were evaluated using participants’ daily diary entries. UPDRS and Clinical Global Impression (CGI) were also measured before and after treatment, and instances of adverse events were monitored.	Participants receiving istradefylline had a significant reduction in awake time spent in the “off” state compared with controls regardless of dosage (*p* = 0.008). Though there were no changes in dyskinesia severity, those receiving istradefylline had significantly increased “on” time with dyskinesia compared to placebo group (*p* = 0.002). There were no differences in changes of UPDRS and CGI scores. The most common adverse event was nausea, though istradefylline was overall well-tolerated.	Istradefylline offers reduced “off” time and increased “on” time with dyskinesia compared with controls as measured by home diary entries.
LeWitt et al. (2008)	In the 6002-US-005 double-blind randomized control trial (RCT) participants with levodopa-treated PD with prominent wearing-off motor fluctuations (*n* = 196) were recruited (172 completed the trial) and randomly assigned to receive either istradefylline 40 mg/day (*n* = 114) or placebo (*n* = 58) for 12 weeks. The primary outcome of interest was change in percentage of daily awake “off” time. Secondary outcomes included “on” time, UPDRS score, and CGI-Improvement of Illness (CGI-I) score. Adverse events were also monitored.	Compared with baseline, participants who received istradefylline 40 mg/day (95% confidence interval, −13.46 to −7.52) had a significantly decreased percentage of daily awake “off” time compared with those who received placebo (95% confidence interval, −7.73–0.31; *p* = 0.007). Daily “on” time increased significantly for the istradefylline 40 mg/day group compared with placebo. No significant differences in UPDRS scores or CGI-I scores were observed. Instances of adverse events were rare and comparable amongst groups.	Istradefylline 40 mg/day was well-tolerated and offered a clinically meaningful reduction in “off” time for levodopa-treated patients with PD with motor complications.
Stacey et al. (2008)	The 6002-US-006 double-blind RCT assigned 347 participants with PD taking levodopa to either istradefylline 20 mg/day (*n* = 152), istradefylline 60 mg/day (*n* = 126), or placebo (*n* = 69) for a duration of 12 weeks. The primary efficacy variable was change in daily OFF time; additional measures included ON time, UPDRS, and CGI. Adverse events were also documented.	Both istradefylline 20 mg/day and 60 mg/day resulted in significant decreased OFF time per day compared with placebo. No differences in UPDRS and CGI scores were observed. Though istradefylline was well-tolerated, the most common adverse events were dyskinesia, nausea, dizziness, and hallucinations.	Istradefylline 20 mg/day and 60 mg/day improve daily OFF rates in patients with levodopa-treated PD.
Mizuno et al. (2010)	In this phase III RCT of 363 participants, subjects were randomly assigned to receive either KW-6002 (istradefylline) 20 mg/day (*n* = 119), 40 mg/day istradefylline (*n* = 125), or placebo (*n* = 119). Primary outcomes of interest included UPDRS Part III and reduction in daily OFF time compared with baseline.	The primary outcome of daily OFF time was reduced from baseline by 1.31 h for the group receiving 20 mg/day istradefylline, 1.58 for the 40 mg/day group, and 0.66 h for the placebo group. The UPDRS Part III subscale score measuring the ON state was reduced by 5.7 points in both intervention groups and 3.7 points in the placebo group. Dyskinesia was the most common adverse event and was reported in 2.5% (3/119) of those receiving placebo, 8.5% (10/118) of those receiving 20 mg/day istradefylline, and 6.4% (8/125) of those receiving 40 mg/day istradefylline.	Istradefylline at 20 mg and 40 mg once daily is well tolerated and effective in relieving wearing-off fluctuations in patients with PD.
Factor et al. (2010)	This open-label, multileft safety and tolerability trial recruited 496 subjects with PD on levodopa to receive open-label istradefylline 40 mg/day for 2 weeks then 20–60 mg/day thereafter with a mean exposure of 25 weeks. Participants in Group I (*n* = 315) were those who completed double-blind treatment with istradefylline within 15 days prior to entering this study. Those in Group II (*n* = 181) were considered washed-out from any prior istradefylline exposure or were naïve to istradefylline. Change in OFF time was the primary outcome of interest.	There was no significant reduction in OFF time for participants who received istradefylline over the course of the trial period. Participants in Group II had a significant decrease in OFF time with consistent improvement in symptoms over a 52 week duration. Due to poor participant retention, this data should be interpreted with caution.	Istradefylline produced a sustained reduction in off time in levodopa-treated PD patients over a 52 week period.
Pourcher et al. (2012)	The KW-6002-US-018 randomized, double-blind, placebo-controlled parallel-group study randomly assigned 610 participants with PD to receive 12 weeks of either istradefylline 10 mg/day (n = 149), 20 mg/day (*n* = 144), 40 mg/day (*n* = 145), or placebo. Outcomes of interest included change in daily awake OFF time, UPDRS Part III score, and medication safety as measured by instances of adverse events.	Interestingly, the percentage of OFF time was comparable across all treatment groups. There was a modest yet significant improvement in UPDRS Part III score in the istradefylline 40 mg/day group compared with placebo. The most common adverse events were dyskinesia and insomnia, and rates were comparable across all groups.	In contrast to prior RCTs, adjunctive istradefylline failed to meet the primary goal of reducing OFF time for patients with PD with motor fluctuations.
Mizuno & Kondo (2013)	A RCT consisting of 373 PD patients were randomized to receive placebo (*n* = 126), 20 mg/day istradefylline (*n* = 123), or 40 mg/day istradefylline (*n* = 124) for a duration of 12 weeks. The primary efficacy variable was change in daily OFF time. The UPDRS Part III score was also evaluated before and after treatment. Instances of adverse events were also evaluated.	Daily OFF time was significantly reduced in the istradefylline 20 mg/day and 40 mg/day groups versus the placebo group; however there was no significant difference in change in daily OFF time between the two istradefylline dose groups. Interestingly, the change from baseline in UPDRS Part III score at the trial endpoint was significantly reduced in the istradefylline 40 mg/day group only. Dyskinesia was the most common adverse event (placebo, 4.0%; istradefylline 20 mg/day, 13.0%; istradefylline 40 mg/day, 12.1%).	Istradefylline reduced daily OFF time for Japanese patients with levodopa-treated PD with motor complications and was generally well-tolerated.
Iijima et al. (2019)	This multileft, open-label, single-group, prospective international study assessed changes in UPDRS Part II/III scores and Freezing of Gait Questionnaire (FOG-Q) in 31 patients with PD treated with istradefylline. Participants received istradefylline 20 mg/day for 4 weeks followed by 20 mg/day or 40 mg/day istradefylline for 8 more weeks for a total of 12 weeks of treatment.	At weeks 4–12, UPDRS Part III gait-related scores significantly decreased with significant improvements in gait, freezing of gait, and postural stability. Significant decreases in UPDRS Part II scores at week 12 reflected improved daily living activities. Additionally, at week 12 there were significant improvements in Freezing of Gait Questionnaire (FOG-Q) and overall movement per 48 h as measured by portable gait rhythm-gram. Adverse events were rare (7/31 patients)	Istradefylline improved gait disorders and improved quality of life in patients with PD complicated with freezing of gait.
Fujioka et al. (2019)	This open-label study investigated the utility of istradefylline in the treatment of postural abnormalities in 21 levodopa-treated patients with PD. MDS-UPDRS Part III score changes, qualitative questionnaires, and sleep scale changes were evaluated after treatment with istradefylline.	The subitem score of posture on the MDS-UPDRS Part III score significantly improved with istradefylline treatment but the changes in score of posture did not correlate with changes in other items on MDS-UPDRS Part III, qualitative questionnaires, or sleep scale changes.	Istradefylline improved posture scores on the MDS-UPDRS Part III.

**Table 2:** Comparative Studies.

Author (Year)	Groups Studied and Intervention	Results and Findings	Conclusions
Betz et al. (2009)	A controlled study using rat models compared istradefylline and tropicamide, a muscarinic antagonist, in the treatment of pimozidine-induced oral tremor.	Istradefylline significantly reduced ventrolateral neostriatum overactivation and improved symptoms of pimozidine-induced oral tremor. Surprisingly, tropicamide increased ventrolateral neostriatum activation yet achieved comparable improvement in oral tremor symptoms.	Through a different mechanism of action, istradefylline is as effective as tropicamide for pimozidine-induced oral tremor.

## References

[1] de Lau L.M., Breteler M.M. (2006). Epidemiology of Parkinson’s disease. Lancet Neurol..

[2] Ray Dorsey E., Elbaz A., Nichols E., Abd-Allah F., Abdelalim A., Adsuar J.C., Ansha M.G., Brayne C., Choi J.Y.J., Collado-Mateo D. (2018). Global, regional, and national burden of Parkinson’s disease, 1990–2016: A systematic analysis for the global burden of disease study 2016. Lancet Neurol..

[3] Vijiaratnam N., Foltynie T. (2020). Therapeutic strategies to treat or prevent off episodes in adults with Parkinson’s Disease. Drugs.

[4] Ascherio A., Schwarzschild M.A. (2016). The epidemiology of Parkinson’s disease: Risk factors and prevention. Lancet Neurol..

[5] Pringsheim T., Jette N., Frolkis A., Steeves T.D.L. (2014). The prevalence of Parkinson’s disease: A systematic review and meta-analysis. Mov. Disord..

[6] Armstrong M.J., Okun M.S. (2020). Diagnosis and treatment of Parkinson disease: A review. JAMA J. Am. Med. Assoc..

[7] Ando Y., Fujimoto K., Ikeda K., Utsumi H., Okuma Y., Oka H., Kamei S., Kurita A., Takahashi K., Nogawa S. (2019). Postural abnormality in Parkinson’s Disease: A large comparative study with general population. Mov. Disord. Clin. Pract..

[8] Obeso J.A., Rodriguez-Oroz M.C., Rodriguez M., Lanciego J.L., Artieda J., Gonzalo N., Olanow C.W. (2000). Pathophysiology of the basal ganglia in Parkinson’s disease. Trends Neurosci..

[9] Moisan F., Kab S., Mohamed F., Canonico M., Le Guern M., Quintin C., Carcaillon L., Nicolau J., Duport N., Singh-Manoux A. (2016). Parkinson disease male-to-female ratios increase with age: French nationwide study and meta-analysis. J. Neurol. Neurosurg. Psychiatry.

[10] Thenganatt M.A., Jankovic J. (2014). Parkinson disease subtypes. JAMA Neurol..

[11] Kenborg L., Lassen C.F., Ritz B., Andersen K.K., Christensen J., Schernhammer E.S., Hansen J., Wermuth L., Rod N.H., Olsen J.H. (2015). Lifestyle, family history, and risk of idiopathic parkinson disease: A large danish case-control study. Am. J. Epidemiol..

[12] Liu R., Guo X., Park Y., Huang X., Sinha R., Freedman N.D., Hollenbeck A.R., Blair A., Chen H. (2012). Caffeine intake, smoking, and risk of parkinson disease in men and women. Am. J. Epidemiol..

[13] Hirsch L., Jette N., Frolkis A., Steeves T., Pringsheim T. (2016). The incidence of Parkinson’s disease: A systematic review and meta-analysis. Neuroepidemiology.

[14] Hussl A., Seppi K., Poewe W. (2013). Nonmotor symptoms in Parkinson’s disease. Expert Rev. Neurother..

[15] Fox S.H., Katzenschlager R., Lim S.Y., Barton B., de Bie R.M.A., Seppi K., Coelho M., Sampaio C. (2018). International Parkinson and movement disorder society evidence-based medicine review: Update on treatments for the motor symptoms of Parkinson’s disease. Mov. Disord..

[16] Muzerengi S., Clarke C.E. (2015). Initial drug treatment in Parkinson’s disease. BMJ.

[17] Fahn S., Oakes D., Shoulson I., Kieburtz K., Rudolph A., Marek K., Seibyl J., Lang A., Olanow C.W., Tanner C. (2004). Levodopa and the progression of Parkinson’s disease. N. Engl. J. Med..

[18] Cabreira V., Soares-da-Silva P., Massano J. (2019). Contemporary options for the management of motor complications in Parkinson’s disease: Updated clinical review. Drugs.

[19] Gray R., Ives N., Rick C., Patel S., Gray A., Jenkinson C., McIntosh E., Wheatley K., Williams A., Clarke C.E. (2014). Long-term effectiveness of dopamine agonists and monoamine oxidase B inhibitors compared with levodopa as initial treatment for Parkinson’s disease (PD MED): A large, open-label, pragmatic randomised trial. Lancet.

[20] Ahlskog J.E., Muenter M.D. (2001). Frequency of levodopa-related dyskinesias and motor fluctuations as estimated from the cumulative literature. Mov. Disord..

[21] Weintraub D., Koester J., Potenza M.N., Siderowf A.D., Stacy M., Voon V., Whetteckey J., Wunderlich G.R., Lang A.E. (2010). Impulse control disorders in Parkinson disease. Arch. Neurol..

[22] Weintraub D., David A.S., Evans A.H., Grant J.E., Stacy M. (2015). Clinical spectrum of impulse control disorders in Parkinson’s disease. Mov. Disord..

[23] Olanow C.W., Stern M.B., Sethi K. (2009). The scientific and clinical basis for the treatment of Parkinson disease (2009). Neurology.

[24] Poewe W., Rascol O., Barone P., Hauser R.A., Mizuno Y., Haaksma L. (2011). Extended-release pramipexole in early Parkinson disease. Neurology.

[25] Sethi K.D., O’Brien C.F., Hammerstad J.P., Adler C.H., Davis T.L., Taylor R.L., Sanchez-Ramos J., Bertoni J.M., Hauser R.A. (1998). Ropinirole for the treatment of early Parkinson disease: A 12-month experience. Arch. Neurol..

[26] Watts R., Jankovic J., Waters C., Rajput A.H., Boroojerdi B., Rao J. (2007). Randomized, blind, controlled trial of transdermal rotigotine in early Parkinson disease. Neurology.

[27] Stowe R.L., Ives N.J., Clarke C., Van Hilten J., Ferreira J., Hawker R.J., Shah L., Wheatley K., Gray R. (2008). Dopamine agonist therapy in early Parkinson’s disease. Cochrane Database Syst. Rev..

[28] Connolly B.S., Lang A.E. (2014). Pharmacological treatment of Parkinson disease: A review. JAMA J. Am. Med. Assoc..

[29] National Collaborating Centre for Chronic Conditions (UK) (2006). Parkinson’s Disease: National Clinical Guideline for Diagnosis and Management in Primary and Secondary Care.

[30] Garcia-Ruiz P.J., Martinez Castrillo J.C., Alonso-Canovas A., Herranz Barcenas A., Vela L., Sanchez Alonso P., Mata M., Olmedilla Gonzalez N., Mahillo Fernandez I. (2014). Impulse control disorder in patients with Parkinson’s disease under dopamine agonist therapy: A multicentre study. J. Neurol. Neurosurg. Psychiatry.

[31] Rabinak C.A., Nirenberg M.J. (2017). Dopamine agonist withdrawal syndrome in Parkinson’s disease. J. Neurol. Sci..

[32] Pondal M., Marras C., Miyasaki J., Moro E., Armstrong M.J., Strafella A.P., Shah B.B., Fox S., Prashanth L.K., Phielipp N. (2013). Clinical features of dopamine agonist withdrawal syndrome in a movement disorders clinic. J. Neurol. Neurosurg. Psychiatry.

[33] Macleod A.D., Counsell C.E., Ives N., Stowe R. (2005). Monoamine oxidase B inhibitors for early Parkinson’s disease. Cochrane Database Syst. Rev..

[34] Caslake R., Macleod A., Ives N., Stowe R., Counsell C. (2009). Monoamine oxidase B inhibitors versus other dopaminergic agents in early Parkinson’s disease. Cochrane Database Syst. Rev..

[35] Tse W. (2006). Optimizing pharmacotherapy: Strategies to manage the wearing-off phenomenon. J. Am. Med. Dir. Assoc..

[36] Seppi K., Ray Chaudhuri K., Coelho M., Fox S.H., Katzenschlager R., Perez Lloret S., Weintraub D., Sampaio C., Chahine L., Hametner E.M. (2019). Update on treatments for nonmotor symptoms of Parkinson’s disease—An evidence-based medicine review. Mov. Disord..

[37] The U.S. Food and Drug Administration Nourianz (istradefylline) Label Highlights of Prescribing Information 2019. https://www.accessdata.fda.gov/drugsatfda_docs/label/2019/761066s000lbl.pdf.

[38] Uchida S.I., Tashiro T., Kawai-Uchida M., Mori A., Jenner P., Kanda T. (2014). The adenosine a2A-receptor antagonist istradefylline enhances the motor response of L-DOPA without worsening dyskinesia in MPTP-treated common marmosets. J. Pharmacol. Sci..

[39] Kanda T., Jackson M.J., Smith L.A., Pearce R.K.B., Nakamura J., Kase H., Kuwana Y., Jenner P. (2000). Combined use of the adenosine A(2A) antagonist KW-6002 with L-DOPA or with selective D1 or D2 dopamine agonists increases antiparkinsonian activity but not dyskinesia in MPTP-treated monkeys. Exp. Neurol..

[40] Koga K., Kurokawa M., Ochi M., Nakamura J., Kuwana Y. (2000). Adenosine A(2A) receptor antagonists KF17837 and KW-6002 potentiate rotation induced by dopaminergic drugs in hemi-Parkinsonian rats. Eur. J. Pharmacol..

[41] Jenner P. (2005). Istradefylline, a novel adenosine A2A receptor antagonist, for the treatment of Parkinson’s disease. Expert Opin. Investig. Drugs.

[42] Dungo R., Deeks E.D. (2013). Istradefylline: First global approval. Drugs.

[43] Schiffmann S.N., Jacobs O., Vanderhaeghen J.-J. (1991). Striatal restricted adenosine A2 receptor (RDC8) is expressed by enkephalin but not by substance P neurons: An in situ hybridization histochemistry study. J. Neurochem..

[44] Cieślak M., Komoszyński M., Wojtczak A. (2008). Adenosine A2A receptors in Parkinson’s disease treatment. Purinergic Signal..

[45] Popoli P., Pèzzola A., Scotti de Carolis A. (1994). Modulation of striatal adenosine A1 and A2 receptors induces rotational behaviour in response to dopaminergic stimulation in intact rats. Eur. J. Pharmacol..

[46] Knebel W., Rao N., Uchimura T., Mori A., Fisher J., Gastonguay M.R., Chaikin P. (2011). Population pharmacokinetic analysis of istradefylline in healthy subjects and in patients with Parkinson’s disease. J. Clin. Pharmacol..

[47] Rao N., Uchimura T., Mori A. (2008). Evaluation of safety, tolerability, and multiple-dose pharmacokinetics of istradefylline in Parkinson’s disease patients [abstract no. PIII-88]. Clin. Pharmacol. Ther..

[48] Rao N., Uchimura T., Mori A. (2008). Evaluation of safety, tolerability, and multiple-dose pharmacokinetics of istradefylline in healthy subjects [abstract no. PIII-89]. Clin. Pharmacol. Ther..

[49] Rao N., Allenby K., Uchimura T., Rao N., Allenby K., Uchimura T. (2007). Steady state administration of istradefylline does not affect the pharmacokinetics of levodopa/ carbidopa [abstract no. 618]. Mov. Disord..

[50] Hickey P., Stacy M. (2012). Adenosine A2A antagonists in Parkinson’s disease: What’s next?. Curr. Neurol. Neurosci. Rep..

[51] Szabó N., Kincses Z.T., Vécsei L. (2011). Novel therapy in Parkinson’s disease: Adenosine A2A receptor antagonists. Expert Opin. Drug Metab. Toxicol..

[52] Jenner P. (2003). A2A antagonists as novel nondopaminergic therapy for motor dysfunction in PD. Neurology.

[53] Pinna A. (2014). Adenosine A2A receptor antagonists in Parkinson’s disease: Progress in clinical trials from the newly approved istradefylline to drugs in early development and those already discontinued. CNS Drugs.

[54] Bara-Jimenez W., Sherzai A., Dimitrova T., Favit A., Bibbiani F., Gillespie M., Morris M.J., Mouradian M.M., Chase T.N. (2003). Adenosine A2A receptor antagonist treatment of Parkinson’s disease. Neurology.

[55] Hauser R.A., Hubble J.P., Truong D.D. (2003). Randomized trial of the adenosine A2A receptor antagonist istradefylline in advanced PD. Neurology.

[56] Mizuno Y., Kondo T. (2013). Adenosine A2A receptor antagonist istradefylline reduces daily OFF time in Parkinson’s disease. Mov. Disord..

[57] Mizuno Y., Hasegawa K., Kondo T., Kuno S., Yamamoto M., Sakou K., Shoji M., Abe T., Nomura H., Maeda T. (2010). Clinical efficacy of istradefylline (KW-6002) in Parkinson’s disease: A randomized, controlled study. Mov. Disord..

[58] Ishibashi K., Miura Y., Wagatsuma K., Toyohara J., Ishiwata K., Ishii K. (2018). Occupancy of adenosine A2A receptors by istradefylline in patients with Parkinson’s disease using 11C-preladenant PET. Neuropharmacology.

[59] LeWitt P.A., Guttman M., Tetrud J.W., Tuite P.J., Mori A., Chaikin P., Sussman N.M. (2008). Adenosine A2A receptor antagonist istradefylline (KW-6002) reduces off time in Parkinson’s disease: A double-blind, randomized, multicenter clinical trial (6002-US-005). Ann. Neurol..

[60] Stacy M., Silver D., Mendis T., Sutton J., Mori A., Chaikin P., Sussman N.M. (2008). A 12-week, placebo-controlled study (6002-US-006) of istradefylline in Parkinson disease. Neurology.

[61] Pourcher E., Huot P. (2015). Adenosine 2A receptor antagonists for the treatment of motor symptoms in Parkinson’s disease. Mov. Disord. Clin. Pract..

[62] Pourcher E., Fernandez H.H., Stacy M., Mori A., Ballerini R., Chaikin P. (2012). Istradefylline for Parkinson’s disease patients experiencing motor fluctuations: Results of the KW-6002-US-018 study. Park. Relat. Disord..

[63] Chen W., Wang H., Wei H., Gu S., Wei H. (2013). Istradefylline, an adenosine A2A receptor antagonist, for patients with Parkinson’s disease: A meta-analysis. J. Neurol. Sci..

[64] Tao Y., Liang G. (2014). Efficacy of adenosine A2A receptor antagonist istradefylline as augmentation for Parkinson’s disease: A meta-analysis of randomized controlled trials. Cell. Biochem. Biophys..

[65] Factor S., Mark M.H., Watts R., Struck L., Mori A., Ballerini R., Sussman N.M. (2010). A long-term study of istradefylline in subjects with fluctuating Parkinson’s disease. Park. Relat. Disord..

[66] Iijima M., Orimo S., Terashi H., Suzuki M., Hayashi A., Shimura H., Mitoma H., Kitagawa K., Okuma Y. (2019). Efficacy of istradefylline for gait disorders with freezing of gait in Parkinson’s disease: A single-arm, open-label, prospective, multicenter study. Expert Opin. Pharmacother..

[67] Uchida S.I., Soshiroda K., Okita E., Kawai-Uchida M., Mori A., Jenner P., Kanda T. (2015). The adenosine A2A receptor antagonist, istradefylline enhances anti-parkinsonian activity induced by combined treatment with low doses of L-DOPA and dopamine agonists in MPTP-treated common marmosets. Eur. J. Pharmacol..

[68] Yabe I., Kitagawa M., Takahashi I., Matsushima M., Sasaki H. (2017). The efficacy of istradefylline for treating mild wearing-off in Parkinson disease. Clin. Neuropharmacol..

[69] Knebel W., Rao N., Uchimura T., Mori A., Fisher J., Gastonguay M.R., Chaikin P. (2012). Population pharmacokinetic-pharmacodynamic analysis of istradefylline in patients with Parkinson disease. J. Clin. Pharmacol..

[70] Suzuki K., Miyamoto T., Miyamoto M., Uchiyama T., Hirata K. (2018). Could istradefylline be a treatment option for postural abnormalities in mid-stage Parkinson’s disease?. J. Neurol. Sci..

[71] Fujioka S., Yoshida R., Nose K., Hayashi Y., Mishima T., Fukae J., Kitano K., Kikuchi H., Tsuboi Y. (2019). A new therapeutic strategy with istradefylline for postural deformities in Parkinson’s disease. Neurol. Neurochir. Pol..

[72] Yasuda Y. (2018). Reversible istradefylline-induced pleurothotonus in a patient with Parkinson’s disease: A case report and literature review. eNeurologicalSci.

[73] Nagayama H., Kano O., Murakami H., Ono K., Hamada M., Toda T., Sengoku R., Shimo Y., Hattori N. (2019). Effect of istradefylline on mood disorders in Parkinson’s disease. J. Neurol. Sci..

[74] Perez-Lloret S., Merello M. (2014). Two new adenosine receptor antagonists for the treatment of Parkinson’s disease: Istradefylline versus tozadenant. Expert Opin. Pharmacother..

[75] Uchida S., Kadowaki-Horita T., Kanda T. (2014). Effects of the adenosine A2A receptor antagonist on cognitive dysfunction in Parkinson’s disease. International Review of Neurobiology.

[76] Xu K., Bastia E., Schwarzschild M. (2005). Therapeutic potential of adenosine A2A receptor antagonists in Parkinson’s disease. Pharmacol. Ther..

[77] Betz A.J., Vontell R., Valenta J., Worden L., Sink K.S., Font L., Correa M., Sager T.N., Salamone J.D. (2009). Effects of the adenosine A2A antagonist KW 6002 (istradefylline) on pimozide-induced oral tremor and striatal c-Fos expression: Comparisons with the muscarinic antagonist tropicamide. Neuroscience.

[78] Salamone J.D., Betz A.J., Ishiwari K., Felsted J., Madson L., Mirante B., Clark K., Font L., Korbey S., Sager T.N. (2008). Tremorolytic effects of adenosine A2A antagonists: Implications for parkinsonism. Front. Biosci..

[79] DeCerce J., Smith L.F., Gonzalez W., Sussman N.M. (2007). Effectiveness and tolerability of istradefylline for the treatment of restless legs syndrome: An exploratory study in five female patients. Curr. Ther. Res. Clin. Exp..

[80] Matsuura K., Tomimoto H. (2015). Istradefylline is recommended for morning use: A report of 4 cases. Intern. Med..

[81] Kitta T., Yabe I., Takahashi I., Matsushima M., Sasaki H., Shinohara N. (2016). Clinical efficacy of istradefylline on lower urinary tract symptoms in Parkinson’s disease. Int. J. Urol..

[82] Navarro G., Borroto-Escuela D.O., Fuxe K., Franco R. (2016). Purinergic signaling in Parkinson’s disease. Relevance for treatment. Neuropharmacology..

[83] Li Z.J., Wu Q., Yi C.J. (2015). Clinical efficacy of istradefylline versus rTMS on Parkinsons disease in a randomized clinical trial. Curr. Med. Res. Opin..

[84] Park A., Stacy M. (2012). Istradefylline for the treatment of Parkinson’s disease. Expert Opin. Pharmacother..

